# Quantitation of 1,4-Dichlorobenzene and Thymol in Beeswax Using Dynamic Headspace Vacuum Transfer in Trap Extraction Prior to Gas Chromatography-Mass Spectrometry

**DOI:** 10.3390/molecules27175367

**Published:** 2022-08-23

**Authors:** Christina Kast, Marion Fracheboud, Pascal Fuchsmann

**Affiliations:** 1 Swiss Bee Research Center, Agroscope, Schwarzenburgstrasse 161, 3003 Bern, Switzerland; 2 Human Nutrition, Sensory Analysis and Flavour, Agroscope, Schwarzenburgstrasse 161, 3003 Bern, Switzerland

**Keywords:** 1,4-dichlorobenzene, thymol, residues, beeswax, dynamic headspace, vacuum transfer in trap extraction, gas chromatography, mass spectrometry

## Abstract

A method based on dynamic headspace vacuum transfer in trap extraction, followed by gas chromatography coupled with a mass spectrometer (DHS-VTT-GC-MS), was validated for the fast quantitation of 1,4-dichlorobenzene (p-dichlorobenzene; PDCB) and thymol residues in beeswax. The quantitation limits (LOQ) were 0.05 mg/kg (PDCB) and 0.25 mg/kg (thymol). Recoveries above 80% were obtained for PDCB concentrations between 0.05 and 10 mg/kg and for thymol concentrations between 0.25 and 200 mg/kg. Analysis of beeswax samples showed a good correlation between the results obtained by DHS-VTT-GC-MS analysis and those of a previous method based on SPE extraction followed by gas chromatography and triple- quadrupole mass spectrometry (GC-MS/MS) (R^2^ = 0.9770 for PDCB and 0.9666 for thymol). However, the sample preparation and chromatography were much shorter using the DHS-VTT-GC-MS method. Forty comb foundations samples produced in Switzerland in 2019 and 2021 were analysed using DHS-VTT-GC-MS. Fourteen samples contained PDCB above the LOQ of 0.05 mg/kg, ranging up to a maximum of 1.53 mg/kg. The mean concentration of the positive samples was 0.22 mg/kg. All samples contained thymol residues ranging between 3.9 and 84.4 mg/kg with mean and median concentrations of 22.7 mg/kg and 17.4 mg/kg. Residue levels of PCDB and thymol in Swiss beeswax were substantially below those measured 20 (PDCB) and 10 (thymol) years ago, respectively.

## 1. Introduction

Residues in hive products originate from products used in beekeeping or enter beehives as environmental contaminants when bees collect nectar, honeydew, pollen, propolis or water. Lipophilic veterinary drugs used in beekeeping for the control of the mite *Varroa destructor* are among the most frequently found residue types in beeswax [1,2,3,4]. Thymol-containing products, such as Apiguard, Thymovar or Api Life Var, are authorised in many countries for mite control [5,6]. The application of such products can lead to thymol residues in beeswax [7]. In Switzerland, thymol-containing products have been marketed for beekeeping since 1996 [7]. Since this time, Swiss beeswax has been regularly monitored for the presence of thymol [8]. For the label programme Bio Suisse and Demeter, the use of thymol is not allowed and a maximal limit of 5 mg/kg is set for thymol in beeswax [9], whereas no such limit exists for beeswax in the common wax cycle outside of these label programmes.

In the past, beekeepers used 1,4-dichlorobenzene (p-dichlorobenzene; PDCB) to protect beeswax from the greater wax moth *Galleria mellonella* and the lesser wax moth *Achroia grisella*. However, PDCB is an insecticide that has never been authorised for use in apicultures. PDCB easily migrates from beeswax into honey. During the years 1997–2002, the cantonal food control authorities in Switzerland detected PDCB in approximately 30% of Swiss honeys [10]. Consequently, Swiss beeswax has been monitored for the presence of PDCB [8,11].

Bogdanov and colleagues developed an extraction procedure for the quantitation of PDCB or thymol in beeswax [7,10]. The original protocol was slightly adapted and allowed simultaneous quantification of PDCB and thymol [8]. However, this extraction procedure is time-consuming, since it is based on solid-phase extraction and includes several freezing steps. Other sample preparation methods described for the determination of thymol in beeswax use distillation [12] or extraction by QuEChERS [13,14].

Recently, a novel method has been developed for volatile compounds in the context of aroma analytics using a dynamic headspace vacuum transfer in trap extraction coupled with a gas chromatography-mass spectrometer (DHS-VTT-GC-MS) [15]. An interface with a vacuum pump with pressure control and commercial in-tube extraction (ITEX) tools allows the detection of volatiles at very low concentrations. Thus far, this method has not been tested for the quantitation of contaminants in food or bee products. A main advantage of adapting such a method for residue analysis in beeswax could be its cost efficiency due to an easy and clean extraction procedure. Hence, the aim of this study was to test whether DHS-VTT-GC-MS allows quantitation of thymol and PDCB residues in beeswax and, if this is the case, to validate the method for quality control of beeswax with respect to these volatiles.

## 2. Results

PDBC and thymol were extracted with methanol in an ultrasonic bath prior to being subjected to DHS-VTT for rapid and efficient extraction of the volatiles from the methanol and injection into the gas chromatograph. The volatiles were separated by gas chromatography and qualified and quantified by mass spectrometry.

### 2.1. Sensitivity and Linearity of the DHS-VTT-GC-MS Method

The limits of detection (LOD) of PCDB and thymol in wax were established using blank wax extract spiked with PDCB and thymol. The LODs were 0.015 mg/kg (PDCB) and 0.1 mg/kg (thymol; Table 1). The calibration curves used for external quantitation were matrix-matched and ranged from 0.0025 mg/L to 2.5 mg/L (PDCB) and from 0.025 mg/L to 25 mg/L (thymol). They showed good linearity (R^2^ = 0.9996 and R^2^ =0.9978, respectively; Table 1).

### 2.2. Validation of the DHS-VTT-GC-MS Method

#### 2.2.1. Quantitation of PDCB and Thymol Using External Calibration

Quantitation of PDCB and thymol was performed by external calibration using calibration curves. For external calibration, the internal standard PDCB-d4 served as a visual injection control, and no correctional factor was applied.

#### 2.2.2. Quantitation of PDCB Using the Internal Standard PDCB-d4

Alternatively, the concentration of PDCB was calculated solely based on the internal standard PDCB-d4, which was added to each sample prior to analysis, such that the final concentration was 0.02 mg/L. The peak area of PDCB in each sample was compared to the peak area of PDCB-d4, which corresponded to 0.02 mg/L. Quantitation of thymol was solely based on external calibration, since deuterated thymol was not available at a reasonable cost.

#### 2.2.3. Recoveries of PDCB and Limits of Quantitation

Recoveries were determined at seven spiking levels. Recoveries of PDCB were close to or above 80% for all but the highest spiked level (Table 2 and Table 3). The limit of quantitation (LOQ) was set at 0.05 mg/kg (Table 1), which was the lowest spiked level with a good recovery. The recovery of PDCB at the LOQ was 89.5% (quantitation using external calibration; Table 2) and 97.5% (quantitation using PDCB-d4; Table 3), respectively. The complete validated range for the analysis of PDCB in wax and the corresponding precision data are summarised in Table 2 (external calibration) and Table 3 (quantitation with PDCB-d4). Precision data for seven concentration levels were calculated from the results obtained by analysing duplicates of spiked samples on eight different days. A single-factor analysis of variance (ANOVA) was performed to estimate the intraday repeatability (variance within the groups) and intermediate precision corresponding to the within-laboratory reproducibility (sum of variance within the groups and between the groups). The precision data obtained with external calibration and those based on quantitation with the internal standard PDCB-d4 suggested that both calibration methods were suitable for the quantitation of PDCB in beeswax.

**Figure 1:**
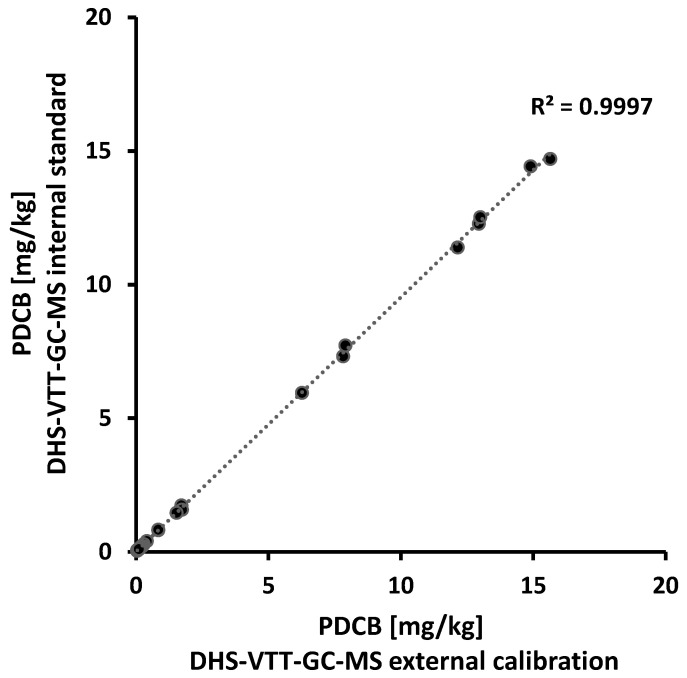
Determination of the PDCB concentration in wax samples (N = 34) by DHS-VTT-GC-MS. Quantitation was achieved through external calibration (x-axis) versus calculation of the PDCB concentration using the internal standard PDCB-d4 (y-axis).

This was further supported by an analysis of 34 wax samples using DHS-VTT-GC-MS. These were individual samples as well as pooled samples from various production years (>LOQ of 0.05 mg/kg), covering most of the concentration range. Both calibration methods were applied for quantification of PDCB and showed a good correlation (R^2^ = 0.9997; Figure 1). Quantitation based on the internal PDCB-d4 (at a final concentration of 0.02 mg/L), however, might be preferred for quantitation of lower PDCB levels, since recoveries and precision values at low spiking levels are above the results obtained by external calibration. Further, quantification using a deuterated standard added to each sample shortens the overall analysis time, since no external calibration curve is necessary.

#### 2.2.4. Recoveries of Thymol and Limits of Quantitation

The thymol recoveries were above 80% for all spiked levels (Table 3). The limit of quantitation (LOQ) was set at 0.25 mg/kg, the lowest validated spiked level with a good recovery (Table 1). The recovery of thymol at the LOQ was 85% (quantitation using external calibration) (Table 4). The complete validated range for thymol in wax and the corresponding precision data (ANOVA analysis) are summarised in Table 4. As the recoveries for most spiking levels were around 85%, the validated DHS-VTT-GC-MS method is suitable for the quantitation of thymol in beeswax. For high spiking levels, the recovery (87.3% at a spiking level of 100 mg/kg) was, however, below the values of the previous method (99.3% at a spiking level of 100 mg/kg [7]).

### 2.3. Analysis of PDCB and Thymol in Commercial Swiss Beeswax

Forty individual commercial beeswax samples produced in 2019 and 2021 by Swiss manufacturers of comb foundations were analysed to determine the range of PDCB and thymol levels in Swiss beeswax. Fourteen foundation samples contained PDCB above the LOQ of 0.05 mg/kg, ranging up to a maximal value of 1.53 mg/kg (Table 5). The mean concentration was 0.077 mg/kg (Table 5), and the mean of the positive samples was 0.22 mg/kg.

All foundations contained thymol residues ranging between 3.9 and 84.4 mg/kg (Table 5). Thus, commercial foundation sheets produced in Switzerland can contain thymol residues of up to 84.4 mg/kg. The mean concentration was 22.7 mg/kg and the median was 17.4 mg/kg (Table 5).

### 2.4. Method Comparison

#### 2.4.1. SPE-GC-MS/MS versus DHS-VTT-GC-MS for Quantitation of PDCB in Beeswax

**Figure 2:**
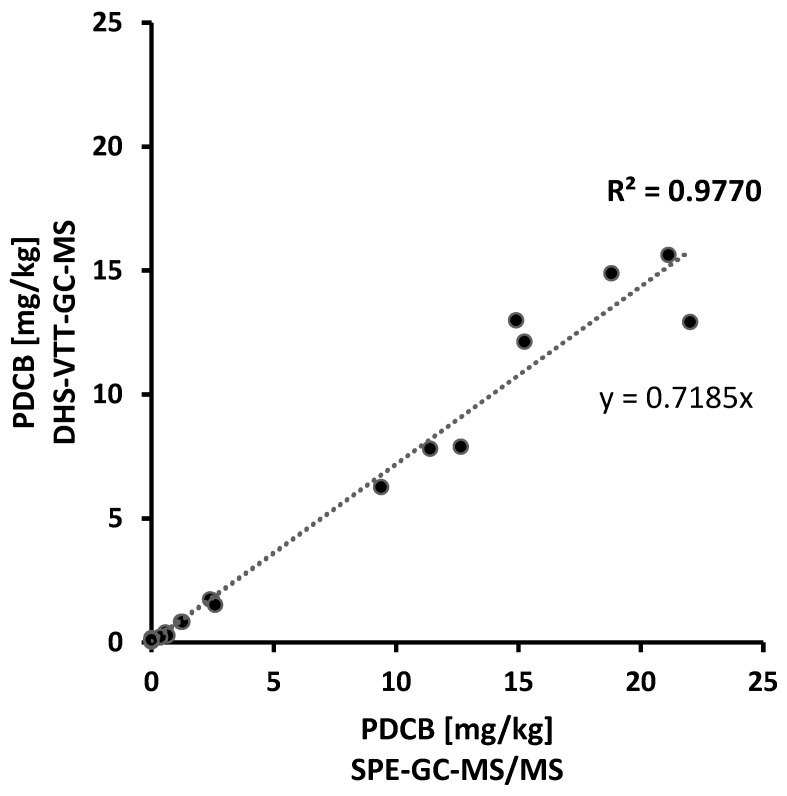
Quantitation of PDCB in wax samples (N = 17) by SPE-GC-MS/MS analysis (x-axis) versus DHS-VTT-GC-MS analysis (y-axis) using external calibration.

The validated DHS-VTT-GC-MS method was compared to a previous SPE extraction procedure followed by GC-MS/MS analysis [8]. Individual samples of recent years, as well as pooled samples of previous years (for a larger concentration range), were analysed using both procedures. Seventeen samples above the previous quantitation limit of 0.37 mg/kg [8] were used to compare the methods. Residue values obtained by SPE-GC-MS/MS analysis were plotted against the values obtained by DHS-VTT-GC-MS (Figure 2). A good correlation (R^2^ = 0.9770) between the two methods was achieved. Thus, both methods gave comparable results. Residue values obtained by the DHS-VTT-GC-MS analysis were, however, consistently below the values obtained by SPE-GC-MS/MS analysis (y = 0.7185x). Most of the deviations might be explained by the application of a correction factor. The recoveries of the previous method using SPE extraction and subsequent GC-MS/MS analysis were between 49% and 57% [8]. To correct for low recoveries, the obtained results were previously multiplied by a correctional factor of 1.85 [8], extrapolating to a recovery rate of 100%. Recoveries using the DHS-VTT-GC-MS method were higher, mostly around 85%, and there was no need for an extrapolation to a 100% recovery rate.

#### 2.4.2. SPE-GC-MS/MS versus DHS-VTT-GC-MS for Quantitation of Thymol in Beeswax

**Figure 3:**
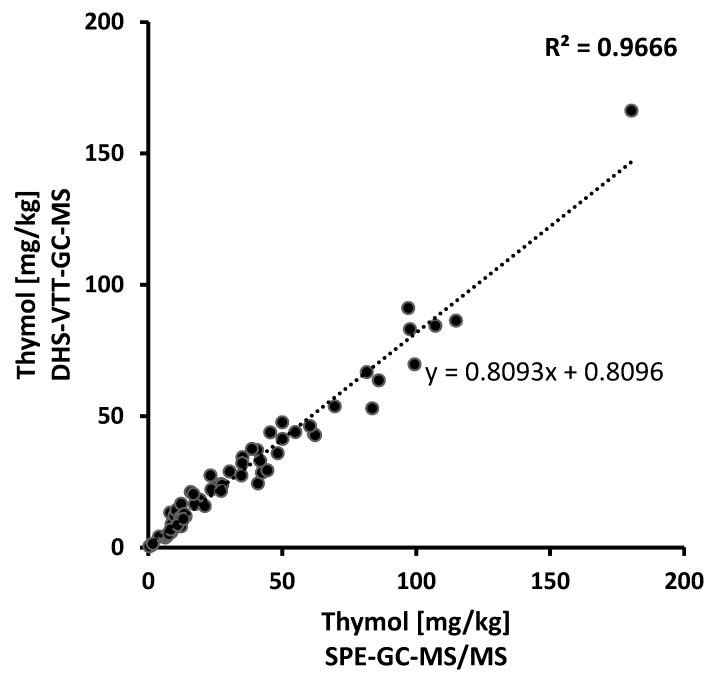
Quantitation of thymol in wax samples (N = 74) by SPE-GC-MS/MS analysis (x-axis) versus DHS-VTT-GC-MS analysis (y-axis).

For the comparison of the methods regarding thymol, residue values of 74 samples above the previous quantitation limit of 0.4 mg/kg [8] were used. Residue values obtained by SPE-GC-MS/MS analysis were plotted against the values obtained by DHS-VTT-GC-MS (Figure 3). A good correlation (R^2^ = 0.9666) between the two methods was achieved. Thus, both methods consistently gave comparable results. Residue values at levels above 50 mg/kg obtained by DHS-VTT-GC-MS analysis were often approximately 20% below the values obtained by SPE-GC-MS/MS analysis (y = 0.8093x + 0.8096). This deviance was probably related to the excellent recovery rate at high spiking levels approaching 100% obtained with the previous SPE extraction procedure and GC-MS/MS analysis (99.3% at a spiking level of 100 mg/kg [7]). The extraction with methanol followed by DHS-VTT-GC-MS produced good recovery rates (87.3% at a spiking level of 100 mg/kg), although they did not reach 100%. Taken together, comparable results were obtained by the two methods, allowing a comparison of the current analysis with the previous PDCB and thymol residue levels measured in Swiss beeswax.

## 3. Discussion

The extraction with DHS-VTT followed by GC-MS analysis allowed quantitation of thymol and PDCB residues in beeswax. The validated method requires minimal sample preparation and allows fast chromatography. Sensitivities and validated concentration ranges are well adapted to the residue levels currently measured in beeswax. The DHS-VTT-GC-MS method allowed measurements at lower PDCB (0.05 mg/kg) and thymol (0.25 mg/kg) levels than the previous SPE-GC-MS/MS method with LOQs for PDCB and thymol of 0.37 mg/kg and 0.40 mg/kg, respectively [8]. Recoveries above 80% were obtained for PDCB concentrations between 0.05 and 10 mg/kg and for thymol concentrations between 0.25 and 200 mg/kg. Two of the main advantages of the validated method are minimal sample preparation and a reduced risk of contamination and clogging of the column by wax components. Thus, the validated method is especially suitable for routine testing.

The vacuum transfer in trap extraction technique has many advantages for qualitative and quantitative measurements of volatile compounds in complex matrices, such as beeswax. First, the technique does not require time-consuming and costly sample preparation, as shown here for the analysis of PDCB and thymol. This limits the consumption of extraction consumables. Apart from a small amount of solvent (here methanol) to dissolve the sample, no further consumables are needed for the extraction. Furthermore, the ITEX extraction equipment can be used for the analysis of several thousand samples without ever having to be changed, thus avoiding the influence of the quality variations of the extraction polymer on the measurements.

Second, the use of commercial ITEX equipment combined with a vacuum pump allows dynamic extraction, which is very efficient, since it avoids reaching an equilibrium of the volatiles between the sample matrix and the trap. Extraction methods under vacuum by combining vacuum extraction with solid-phase microextraction (SPME) have already been described previously [16,17,18,19,20,21]. In contrast to the previously described SPME methods, DHS-VTT extraction is substantially more efficient than a manual vacuum procedure because DHS-VTT extraction is automated, hence allowing for the analysis of large analytical sequences without having to manually vacuum each sample. Thus, DHS-VTT extraction saves time and, most importantly, substantially increases the reproducibility of the extractions.

Third, the solvent is completely evaporated, and the sample is dried during the extraction, which implies that the volatiles are totally concentrated in the ITEX needle containing the extraction polymer. This so-called “total extraction” allows the injection of volatile compounds into the injector without solvent or solid impurities that could clog the separation column. With respect to the many advantages of DHS-VTT, it could be interesting to adapt this method for measuring other volatile contaminants in beeswax.

Analysis of commercial beeswax samples showed that the results obtained by the validated DHS-VTT-GC-MS method compare well with the results from the previous method using SPE followed by GC-MS/MS analysis as described by Bogdanov and colleagues [7,8,10]. Thus, future results obtained by analysis using DHS-VTT-GC-MS can easily be compared to the previous PDCB and thymol residue levels measured in Swiss beeswax [8,11]. Further, the recoveries of PDCB using the DHS-VTT-GC-MS method were significantly higher when compared to the previous GC-MS/MS method (49–57% [8]), in which part of the volatile PDCB is probably lost during the sample preparation with SPE. To correct for the low recoveries of the previous method, the results were multiplied by a correctional factor of 1.85 [8], a procedure that is not necessary for the newly validated method, since recoveries for all but the highest spiked level of PDCB were above 80%. In this respect, the validated extraction procedure in combination with DHS-VTT-GC-MS is superior to the previous SPE extraction followed by GC-MS/MS analysis [8].

Previously, pooled samples were analysed to monitor PDCB and thymol levels in Swiss beeswax [8]. Individual foundation samples were not included, since the previous extraction procedure using SPE and several freezing steps, as well as the chromatographic procedure, were substantially more time-consuming. The short sample preparation procedure and shorter chromatography of the validated method using DHS-VTT-GC-MS allowed us to now evaluate the range of residue levels that can be expected in individual foundation samples. Thirty-five percent of the individual foundation samples from 2019 or 2021 contained PDCB, with a mean of 0.22 mg/kg, while 65% of the samples contained no PDCB above the quantitation limit of 0.05 mg/kg. Hence, the current residue levels are far below the values previously measured in Swiss beeswax produced during the years 1994–2002, when the annual average PDCB residue levels in Swiss beeswax were between 4.1 and 10.9 mg/kg [11]. Occasionally, higher values in recent years (up to a maximal value of 1.53 mg/kg; this study) might be caused by recycled wax that has been stored for several years, when PDCB was more commonly used, before the beekeeper brought the wax to the producer of the foundation sheets.

All foundation samples contained thymol residues. The mean concentration was 22.7 mg/kg, which is substantially below the annual average thymol residue levels measured in Swiss foundation sheets produced in 2009 (87.5 mg/kg [8]). Thus, Swiss beekeepers use thymol-containing products less frequently than in the early years of authorisation [8]. Nowadays, the majority of beekeepers use products based on formic acid for mite control. The effect of formic acid is faster, since formic acid acts on the phoretic mites as well as the mites in the cells, while thymol acts solely on phoretic mites. This development is in contrast to the beekeeping practices of some other countries with a growing interest in using thymol for the mite treatment of honeybee colonies [14]. Due to the regular treatment of the bee colonies, thymol can accumulate in the beeswax and subsequently diffuse from wax into honey. Thus, high thymol concentrations in wax may affect the taste of honey. The organoleptic perception threshold value for thymol in honey is around 1.1 mg/kg [22]. Previous studies have shown that thymol levels in wax up to 500 mg/kg do not lead to thymol concentrations in honey above the threshold value of 1.1 mg/kg [7,8]. Hence, the currently measured thymol concentrations in Swiss beeswax, up to a maximum of 84 mg/kg, are far below the level of 500 mg/kg and are thus unlikely to pose a problem for honey quality.

## 4. Materials and Methods

### 4.1. Materials

We obtained 1,4-dichlorobenzene (C12372000), 1,4-dichlorobenzene-D4 (DER-C12372100), and thymol (C17575200) from LGC Standards GmbH (Wesel, Germany). The solvent methanol CHROMASOLV™ (34860-1L) was from Sigma-Aldrich (Buchs, Switzerland).

### 4.2. Spiked Wax Samples

The beeswax used as a blank wax for enrichment was obtained from our own apiaries situated at various locations in the cantons of Bern and Fribourg in Switzerland (wax of the year 2020). This beeswax has never been exposed to PDCB or mite treatment using thymol-containing products. The wax was analysed for residue levels of PDCB and thymol using the method previously described [8]. It contained no measurable PDCB or thymol levels above the detection limits of 0.370 and 0.400 mg/kg, respectively [8]. Since PDCB and thymol are volatile compounds, special measures were taken to ensure that these substances did not evaporate during the fortification process. For the various spiking levels, PDCB and thymol were weighed into small honey jars (60 mL) that could be tightly closed. Next, 12 g wax was melted in a water bath at 80 °C in a separate jar. The wax was then added quickly to each jar containing PDCB and thymol, the lid of the jar immediately closed, and the sample was shaken by hand. To allow a precise calculation of each concentration level, the precise amount of wax added to each sample was determined by weighing the samples after the addition of wax. Subsequently, the wax was melted once more at 80 °C for 5 min to ensure a homogeny of the sample. The resulting seven spiking levels for PDCB were 0.050, 0.098, 0.500, 0.980, 1.96, 9.85, and 19.6 mg/kg, and the seven spiking levels for thymol were 0.246, 0.499, 4.89, 9.96, 49.0, 98.5, and 196 mg/kg.

### 4.3. Beeswax Samples

The samples were obtained from various commercial manufacturers of foundations in Switzerland participating in a long-term study on acaricide residues in Swiss beeswax [8]. Since 1999, these manufacturers have collected wax samples from each production lot produced every alternate year. The manufacturer sent all samples to the Swiss bee research centre, where the samples were stored in the dark in a deep-freezer at −20 °C. Representative annual samples from each manufacturer (pooled samples) were prepared in proportion to the weight of each production lot [8]. The samples chosen for the validation of the new method comprised the described spiked samples, as well as commercial samples. They included 10 pooled samples from the years 2002 to 2011 at a variety of concentration levels, 10 pooled samples of the year 2021, as well as 3 samples of the years 2021 or 2022 from biological apiaries. Additionally, 40 individual samples from the production years 2019 (N = 20) and 2021 (N = 20) were randomly chosen from various manufacturers to investigate the range of the current PDCB and thymol levels in Swiss beeswax. Apart from the analysis of these samples with the method described in the current study, all samples were also analysed by a previous method developed by Bogdanov and colleagues [7,10], which has been slightly adapted in recent years [8]. The samples from the years 2002 to 2011 were reanalysed for this study to ensure that residue levels were not affected by storage.

### 4.4. Sample Preparation

To determine PDCB and thymol, 200 mg of wax was extracted with 2 mL of methanol for 90 min in an ultrasonic bath (VWR, Schlieren, Switzerland) with maximal power. During this time, the samples were shaken three times by hand at regular intervals. Subsequently, the samples were centrifuged in a DuPont Sorvall RC5C (Digitana AG, Horgen, Switzerland) centrifuge for 15 min at 15,000 U/min (27,847 g). Next, the supernatant was decanted, and 100 µL of the supernatant was placed in a 20 mL headspace vial for analysis. Prior to analysis, 10 µL of PDCB-d4 in methanol at a concentration of 0.2 mg/L was added to the samples as an internal standard. The vials were then hermetically sealed with a silicon/Teflon crimp cap.

### 4.5. Extraction of Volatile Compounds

PDCB and thymol were extracted by DHS-VTT [15] using an ITEX-2 Trap packed with Tenax TA, 80/100 mesh/Carbosieve S III, 60/80 mesh (BGB Analytik AG, Böckten, Switzerland) under a vacuum of 15 mbar (vacuum pump Büchi V-300, Flawil, Switzerland). Prior to extraction, the trap was preconditioned at 300 °C for 60 min. The sample was incubated at 80 °C for 5 min, and the volatiles were extracted from the samples at 80 °C for 5 min under agitation, while the syringe temperature was held at 60 °C and the trap at 30°C. Subsequently, the volatiles were desorbed with a helium flow at 1.17 bar (17 psi) for 2 min at a temperature of 300 °C in the split/splitless injector SSL.

### 4.6. GC-MS Analysis of PDCB and Thymol

Gas chromatography analysis was performed on a gas chromatograph coupled to a mass spectrometer GC-MSD Agilent 7890B/5977B (Gerstel AG, Sursee, Switzerland) and equipped with an autosampler CTC PAL RSI (CTC Analytics AG, Zwingen, Switzerland). An analytical capillary column (OPTIMA FFAP-plus, 320 µm i.d., 1 µm film thickness, Machery-Nagel AG, Oensingen, Switzerland) of 30 m was used for separation. The injection was performed in an SSL in pulsed split mode at a ratio of 20:1 for 3 min. (helium total flow 18.063 mL/min, injection pulsed pressure: 16 psi) at a temperature of 250 °C. The gas chromatograph was operated at a constant column flow of 1.8 mL/min (22 cm/s). The temperature programme started at 40 °C and increased at 10 °C/min to 230 °C, where it was held for 6 min. The total runtime was 25 min. The mass spectrometer was operated in SIM acquisition mode. The temperature of the transfer line and the source was 230 °C, and the ionisation energy was 70 eV operated in positive mode. The mass-to-charge ratios m/z 146 and m/z 135 were used for quantitation of PDCB and thymol, respectively, while the mass-to-charge ratios m/z 111 and 148, as well as m/z 91 and 150 served for identification (Appendix A) together with the retention indices of 1497 for PDCB and 2162 for thymol.

Quantitation was achieved through external calibration using seven concentrations ranging from 0.0025 mg/L to 2.5 mg/L (PDCB) and 0.025 mg/L to 25 mg/L (thymol). The calibration solutions were prepared in blank matrix extract to compensate for the matrix effects. The concentrations of PDCB and thymol in the samples were calculated based on the linear regression (1/x) of the calibration samples using Agilent MassHunter quantitative software Version B.08.00 (Basel, Switzerland). The detector had linear responses for all spiking levels (R2 = 0.9996 and R2 = 0.9978). For concentrations of PDCB and thymol calculated based on the linear regression, the area of PDCB-d4 (m/z 152) was used solely for visual inspection of the injection, but no correctional factor was calculated. Alternatively, the concentration of PDCB in the samples was calculated using the internal standard PDCB-d4 (area of the peak at m/z 152).

The limit of detection (LOD) was experimentally determined using spiked blank wax extracts. The beeswax used as blank wax contained traces of PDCB and thymol that were not detectable using the previous method but nevertheless detectable using the newly developed method. Hence, the LODs were set at levels where the signal was approximately 3-fold above the level in blank extract, for PDCB at 0.0015 mg/L (or 0.015 mg/kg in wax) and for thymol at 0.01 mg/L (or 0.1 mg/kg in wax), respectively. The recovery of PDCB was tested at seven spiking levels (0.050, 0.098, 0.500, 0.980, 1.96, 9.85, 19.6 mg/kg) with eight repetitions each performed in duplicates on various days within 2 weeks.

The recovery of thymol was tested at seven spiking levels (0.246, 0.499, 4.89, 9.96, 49.0, 98.5, 196 mg/kg) with eight repetitions each performed in duplicates on various days within 2 weeks. The limits of quantitation (LOQ) were defined as the lowest validated spike level, where the recoveries were between 80% and 120%. The mean and median values for PDCB and thymol in individual wax samples were calculated across individual foundation samples, taking values below the LOQ as 0.

## Figures and Tables

**Table 1:** Detection and quantitation limits, and the validated range for quantitation of PDCB and thymol in beeswax using DHS-VTT-GC-MS analysis.

	LOD ^1)^ [mg/kg]	LOQ ^2)^ [mg/kg]	Validated Range [mg/kg]	Ex. Calibration [mg/L]	Linearity R ^2), 3)^
PDCB	0.015	0.050	0.050–25	0.0025–2.5	0.9996
Thymol	0.10	0.25	0.25–250	0.025–25	0.9978

^1)^ limit of detection (LOD). ^2)^ limit of quantitation (LOQ). ^3)^ correlation R^2^ of the linear regression.

**Table 2:** Recoveries, repeatability, and intermediate precision obtained by DHS-VTT-GC-MS analysis of PDCB in beeswax using external quantitation.

Spiking Level [mg/kg]	N ^1)^	Mean [mg/kg]	s_r_^2)^ [mg/kg]	RSD_r_^3)^ [%]	s_I_^4)^ [mg/kg]	RSD_I_^5)^ [%]	Recovery [%]
0.050	16	0.045	0.002	4.3	0.007	15.6	89.5
0.098	16	0.086	0.004	4.4	0.008	8.9	87.5
0.500	16	0.414	0.010	2.4	0.026	6.2	83.0
0.980	16	0.846	0.023	2.7	0.066	7.8	86.3
1.96	16	1.67	0.05	2.9	0.10	6.2	84.8
9.85	16	8.37	0.14	1.7	0.75	8.9	85.0
19.6	16	14.8	0.60	4.1	1.8	12.0	75.3

^1)^ Number of repetitions (N). ^2)^ Repeatability(s_r_). ^3)^ Relative standard deviation of repeatability (RSD_r_) expressed as a percentage of the mean. ^4)^ Intermediate precision (s_I_) corresponding to within-laboratory reproducibility. ^5)^ Relative standard deviation of intermediate precision (RSD_I_) expressed as a percentage of the mean.

**Table 3:** Recoveries, repeatability, and intermediate precision obtained by DHS-VTT-GC-MS analysis of PDCB in beeswax using the internal standard PDCB-d4 for quantitation.

Spiking Level [mg/kg]	N ^1)^	Mean [mg/kg]	s_r_^2)^ [mg/kg]	RSD_r_^3)^ [%]	s_I_^4)^ [mg/kg]	RSD_I_^5)^ [%]	Recovery [%]
0.050	16	0.049	0.001	2.3	0.003	5.2	97.5
0.098	16	0.088	0.004	4.6	0.006	6.9	89.7
0.500	16	0.405	0.009	2.2	0.013	3.3	81.1
0.980	16	0.784	0.022	2.8	0.034	4.3	80.0
1.96	16	1.60	0.05	2.9	0.06	3.9	81.6
9.85	16	7.75	0.10	1.3	0.34	4.3	78.7
19.6	16	14.1	0.6	3.9	1.1	7.6	72.0

^1)^ Number of repetitions (N). ^2)^ Repeatability (s_r_). ^3)^ Relative standard deviation of repeatability (RSD_r_) expressed as a percentage of the mean. ^4)^ Intermediate precision (s_I_) corresponding to within-laboratory reproducibility. ^5)^ Relative standard deviation of intermediate precision (RSD_I_) expressed as a percentage of the mean.

**Table 4:** Recoveries, repeatability, and intermediate precision obtained by DHS-VTT-GC-MS analysis of thymol in beeswax using external quantitation.

Spiking Level [mg/kg]	N ^1)^	Mean [mg/kg]	s_r_^2)^ [mg/kg]	RSD_r_^3)^ [%]	s_I_^4)^ [mg/kg]	RSD_I_^5)^ [%]	Recovery [%]
0.246	16	0.209	0.026	12.4	0.051	24.3	85.0
0.499	16	0.421	0.055	13.0	0.063	14.9	84.3
4.89	16	4.17	0.30	7.2	0.34	8.2	85.2
9.96	16	9.22	0.71	7.8	1.25	13.5	92.6
49.0	16	43.9	2.0	4.6	3.9	8.8	89.7
98.5	16	86.0	4.7	5.5	5.6	6.6	87.3
196	16	165	13.8	8.3	20.5	12.4	84.1

^1)^ Number of repetitions (N). ^2)^ Repeatability (s_r_). ^3)^ Relative standard deviation of repeatability (RSD_r_) expressed as a percentage of the mean. ^4)^ Intermediate precision (s_I_) corresponding to within-laboratory reproducibility. ^5)^ Relative standard deviation of intermediate precision (RSD_I_) expressed as a percentage of the mean.

**Table 5:** Mean, median, minimal, and maximal values of PDCB and thymol in commercial Swiss beeswax foundation samples (N = 40) produced in 2019 and 2021.

	N ^1)^	Mean [mg/kg]	Median [mg/kg]	Minimal Value [mg/kg]	Maximal Value [mg/kg]	Number of Positive Samples
PDCB	40	0.077	<LOQ	<LOQ	1.53	14
Thymol	40	22.7	17.4	3.9	84.4	40

^1)^ Number of samples (N).

**Figure A1:**
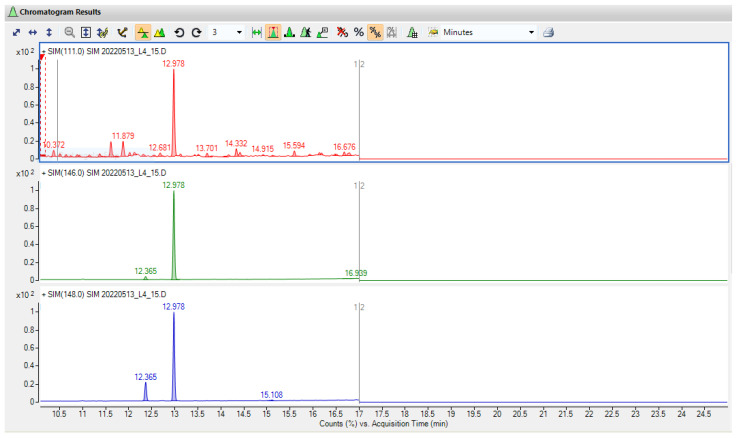
Chromatogram (SIM mode) of PDCB in beeswax. Quantitation ion m/z 146 and qualitative ions m/z 111 and 146.

**Figure A2:**
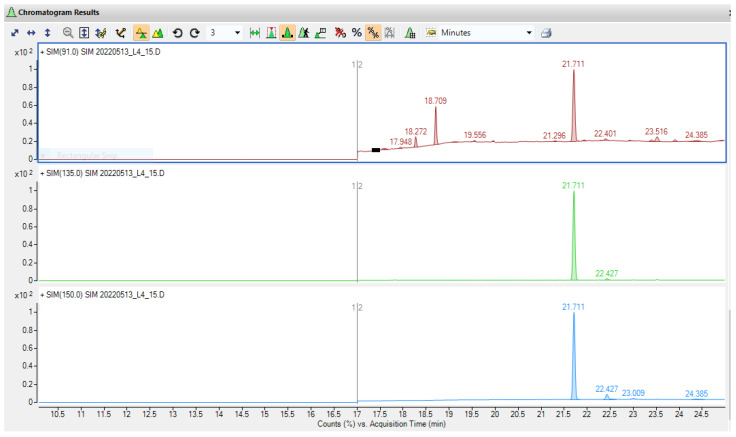
Chromatogram (SIM mode) of thymol in beeswax. Quantitation ion m/z 135 and qualitative ions m/z 150 and 91.

## Data Availability

Not applicable.

## Sample Availability

Samples are not available from the authors.

## Appendix A

Examples of chromatograms of a beeswax sample containing PDCB and thymol: SIM mode, retention times 12.978 min and 21.711 min., respectively. The mass-to-charge ratio m/z 146 was used for quantitation of PDCB, while the mass-to-charge ratios m/z 111 and 148 served for identification. The mass-to-charge ratio m/z 135 was used for quantitation of thymol, while the mass-to-charge ratios m/z 91 and 150 served for identification.
